# Molecular Characterization of *Salmonella* Serovars Anatum and Ealing Associated with Two Historical Outbreaks, Linked to Contaminated Powdered Infant Formula

**DOI:** 10.3389/fmicb.2016.01664

**Published:** 2016-10-21

**Authors:** Lynda Gunn, Sarah Finn, Daniel Hurley, Li Bai, Ellen Wall, Carol Iversen, John E. Threlfall, Séamus Fanning

**Affiliations:** ^1^UCD-Centre for Food Safety, School of Public Health, Physiotherapy and Sports Science, University College DublinDublin, Ireland; ^2^Key Laboratory of Food Safety Risk Assessment, Ministry of Health, China National Center for Food Safety Risk AssessmentBeijing, China; ^3^Health Protection AgencyLondon, UK; ^4^Institute for Global Food Security, Queen's University BelfastBelfast, UK

**Keywords:** PFGE, whole genome sequencing, *Salmonella* pathogenicity islands, core genome, infection model

## Abstract

Powdered infant formula (PIF) is not intended to be produced as a sterile product unless explicitly stated and on occasion may become contaminated during production with pathogens such as *Salmonella enterica*. This retrospective study focused on two historically reported salmonellosis outbreaks associated with PIF from the United Kingdom and France, in 1985 and 1996/1997. In this paper, the molecular characterization of the two outbreaks associated *Salmonella* serovars Anatum and Ealing is reported. Initially the isolates were analyzed using pulsed-field gel electrophoresis (PFGE), which revealed the clonal nature of the two outbreaks. Following from this two representative isolates, one from each serovar was selected for whole genome sequencing (WGS), wherein analysis focused on the *Salmonella* pathogenicity islands. Furthermore, the ability of these isolates to survive the host intercellular environment was determined using an *ex vivo* gentamicin protection assay. Results suggest a high level of genetic diversity that may have contributed to survival and virulence of isolates from these outbreaks.

## Introduction

Infant foods, such as baby cereals and powdered infant milk formulae (PIF), can act as vectors for pathogenic microorganisms of importance to human health as technology is currently unavailable to manufacture these foods as sterile products. Thus, despite the implementation of good manufacturing practices PIF may, on occasion, become contaminated with pathogens during production. Those pathogens that present the greatest threat to infant health include *Cronobacter* species (formerly known as *Enterobacter sakazakii*) and *Salmonella enterica, two* bacterial genera designated by the World Health Organisation (WHO) as Class A pathogens. Following the consumption and ingestion of contaminated foods, clinical signs of infection include gastroenteritis which can progress to bacteraemia and meningitis (Cahill et al., 2008). Numerous *Salmonella* outbreaks associated with contaminated infant foods have been documented since the 1950s and 1960s (Brouard et al., 2007).

In this study two *Salmonella enterica* serovars implicated in PIF outbreaks were investigated. The first outbreak occurred in 1985, when *Salmonella* Ealing, was identified and linked to cases of salmonellosis in the United Kingdom. Some 70 individuals were affected, the majority of these being infants. A subsequent investigation showed low numbers of *Salmonella* organisms in the PIF (~1.6 CFU/450 g) making their detection challenging during routine testing of the food product (Rowe et al., 1987). A second outbreak was reported between 1996/1997 and this was associated with PIF contaminated with *S*. Anatum. On this occasion, the outbreak centered in the United Kingdom and France (Threlfall et al., 1998). Public Health England reported an increase in *S*. Anatum isolations from children less than 1 year old and due to the young age of these patients infant food was suspected to have been responsible for the transmission of the aetiological agent.

This study reports the molecular characterization of *Salmonella enterica* serovars Anatum and Ealing, which were connected to the referenced outbreaks through contaminated PIF. Isolates were initially characterized by macrorestriction-based DNA fingerprinting and later further analyzed by whole genome sequencing (WGS), to observe the genetic diversity of these outbreaks and compare them to other *Salmonella* serovars. Infection assays were performed for two selected isolates from each of these outbreaks. The subsequent analysis focused on *Salmonella* pathogenicity Island (SPI) comparison as well as bacterial survival in *ex-vivo* infection models, to observe potential associations between survival and genetic diversity.

## Materials and methods

### Bacterial culture

Thirty seven bacterial isolates were included in this study comprising of 12 *S*. Anatum and 25 *S*. Ealing. All were stored on beads at −80°C and sub-cultured on tryptone soya agar (TSA) plates at 37°C when required.

### Molecular macrorestriction digest by *Xba*1 and *Spe*1

PFGE pulsotypes were obtained for all isolates following digestion with *Xba*1 restriction enzyme as previously described by Ribot et al. (2006). Isolates that produced indistinguishable patterns with this enzyme were subsequently reanalyzed using a second enzyme, *Spe*I as described by Zheng et al. (2007). Visualization of DNA profiles was carried out using Gel Logic 1500 imaging system. The TIFF files were imported in BioNumerics v.5.1 and dendrograms constructed using the UPGMA and DICE algorithms with 1.0% optimization and 1.5% tolerance.

### Whole genome sequencing and comparative phylogenetic analysis of core *Salmonella* genomes

Whole genome sequencing of isolates was performed using the Illumina MiSeq platform. Library preparation was performed using Nextera XT kit (v3 chemistry) according to manufacturer's instructions producing 300 bp paired end reads. Subsequent raw sequence data was assessed using FastQC. The reads were error corrected using the BFC algorithm before a relaxed quality trim using a sliding window as implemented in Trimmomatic v0.33 (Bolger et al., 2014; Li, 2015). *De novo* genome assemblies were produced using SPAdes assembler v3.6.2 using the default *k-mer* size selection for 300 bp reads with careful mode enabled (used for mismatch error, indel and error correction; Bankevich et al., 2012).

For core genome analysis, comparisons were selected by online BLAST similarity searches of the largest contiguous sequence from *S*. Anatum CFS0056 and *S*. Ealing CFS0080 assemblies. Other serovars were included as they have been associated with food-borne outbreaks previously. Protein sequences from all strains identified by annotation with Prokka (version 1.11) were clustered using the pan-genome pipeline Roary (version 3.6.2) (Seemann, 2014; Page et al., 2015). Visualization of the pan-genome was carried out using Anvi'o (version 2.0.2) (Eren et al., 2015).

### Comparative analysis of selected *Salmonella* pathogenicity islands (SPI)

The differences in SPI associated genes from SPI-1 through -5 were investigated as previously described using standalone BLAST+ v2.4 and comparing these against the corresponding loci from *S*. Typhimurium ST4/74 as the reference genome^1^. The resulting nucleic acid sequences were converted to amino acid sequences using Biopython (version 1.66) and the percentage similarity of the SPI proteins from *S*. Anatum and *S*. Ealing when compared against the reference were determined using the Needleman-Wunsch alignment with default settings through the EMBOSS analysis software (version 6.6.0) (Needleman and Wunsch, 1970). The raw sequencing data and *de novo* assemblies have been deposited at the Sequence Read Archive/GenBank for *S*. Anatum CFS0056 (SRP081283/SAMN05560363) and *S*. Ealing CFS0080 (SRP081283/SAMN05560364).

### *Ex vivo* gentamicin protection assay

A gentamicin protection assay was used to observe whether the isolates could survive phagocytosis using *S*. Typhimurium ST4/74 as the reference strain adapted from protocols as described previously (Lathrop et al., 2015). Briefly THP-1 monocytes were grown in antibiotic-free RPMI 1640 media (Sigma-Aldrich) supplemented with 10% [v/v] heat inactivated FBS and incubated at 37°C with 5% CO_2_. Cells were detached with 1% Triton X-100 and seeded at a density of 1 × 10^5^ cells/mL per well in 24 well-plates. Monocytes were differentiated to adherent macrophages by supplementing media with 20 ng/mL phorbol 12-myristate 13-acetate (PMA) for 5 days.

Prior to infection, bacterial isolates were diluted in complete media to 1 × 10^6^ cells/mL for a MOI of 10:1 and incubated at 37°C for 1 h. Macrophages were washed with 1 mL Hank's Balanced Salt Solution (HBSS), three times, before 1 mL of the bacterial suspension, was added to each well; 1 mL of complete media was added to control wells. The plates were centrifuged at 300 × g for 5 min and incubated for 1 h to allow for phagocytosis. Following phagocytosis, the cells were washed with 1 mL HBSS three times. To kill exposed bacteria 1 mL complete media supplemented with 100 μg/mL gentamicin was added to each well before incubation for 1 h. After the wells were washed with 1 mL HBSS. A final 1 mL of complete media supplemented with 20 μg/mL gentamicin was then added to each well before incubation at for the desired time points. At each time point cells were washed three times with 1 mL HBSS, after 1 mL 1% [v/v] Triton X-100 Phosphate Buffered Saline (PBS) solution was added to the cells and incubated at room temperature for 10 min. The resulting supernatants were decimally diluted in PBS, 100 μL aliquots of the dilutions were plated onto LB agar and incubated for 18 h at 37°C before enumeration.

## Results

### Molecular macrorestriction digest by *Xba*1 and *Spe*1

**Figure 1:**
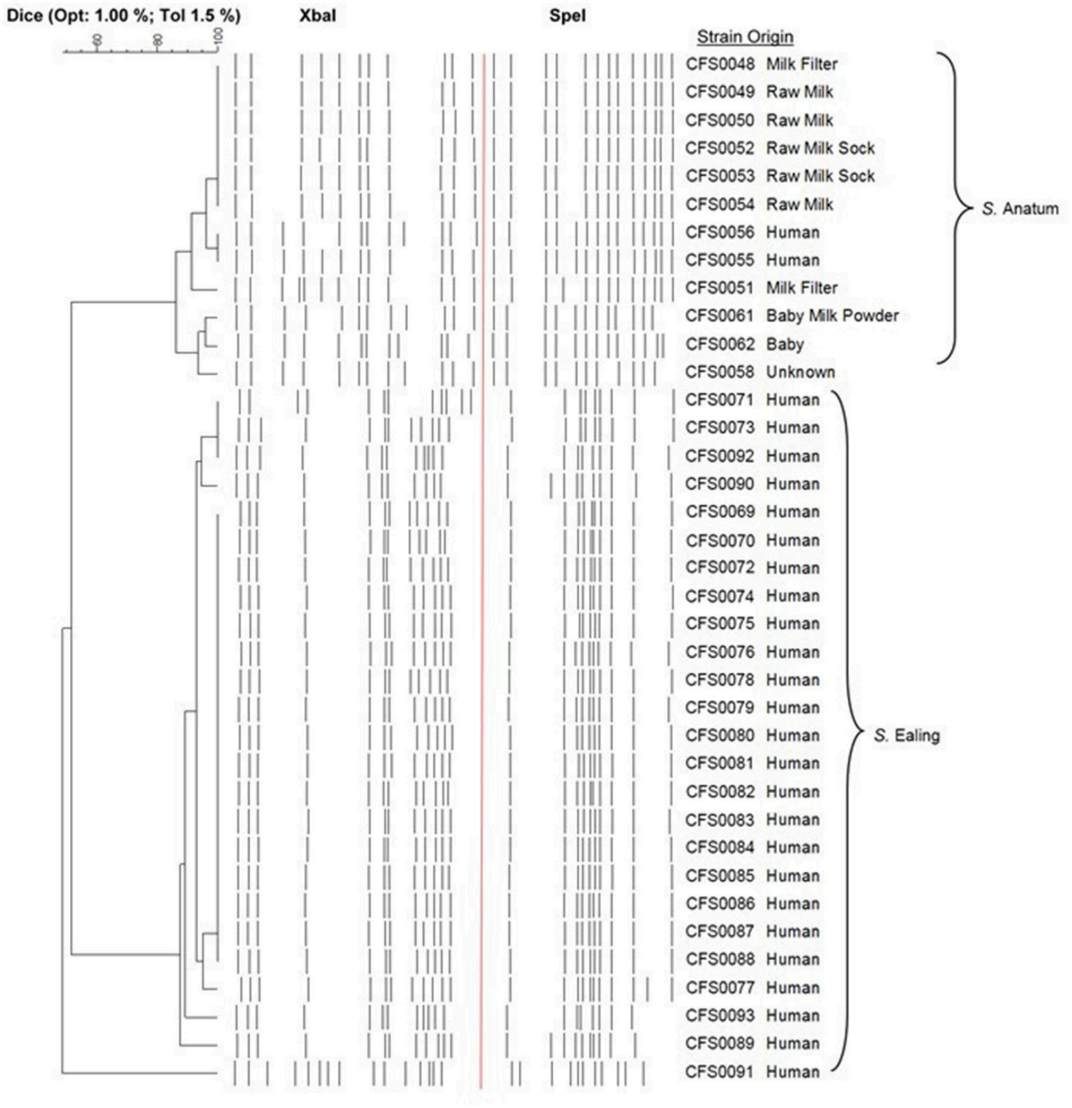
**Dendrogram of combined pulsotype profiles following macrorestriction enzyme digestion with ***Xba***1 and ***Spe***1, of genomic DNA from ***S***. Anatum and ***S***. Ealing isolates**.

The pulsotypes of all 37 isolates were investigated to assess the genetic relationship(s) between those *Salmonella* isolates linked with the two outbreaks in this study. A dendrogram based on the combined pulsotypes from *Xba*1 and *Spe*1 digest representing serovars Anatum and Ealing, is shown in Figure 1. In the case of *S*. Ealing, the pulsotype of the isolate denoted as CFS0091 a non-outbreak strain was notably different (≤7 band difference; when digested with *Xba*1 or *Spe*1) when compared to the other pulsotypes obtained for other members of this serovar (estimated at ~50% similarity). The majority of *S*. Ealing from the outbreak have very similar pulsotypes with the exception of isolates CFS0089, -0093, -0077, -0071, -0073, -0092, and -0090 which displayed more diverse profile (1–3 band differences).

For *S*. Anatum isolates CFS0048-0050 and CFS0052-0056 originally cultured from equipment and clinical cases, these produced very similar pulsotypes, displaying a high degree of similarity (>95%), suggesting a potential epidemiological link. Other isolates from the same serovar showed differences in their restriction patterns (2–4 band difference) with less similarity to clinical isolates (85–90% similarity) making a link less probable.

### Determination of the whole genome sequences for *Salmonella* anatum CFS0056 and ealing CFS0080

Due to the indistinguishable nature of these outbreaks based on their pulsotypes as shown in the PFGE dendrogram (Figure 1), two representative isolates from these PIF outbreaks, *S*. Anatum CFS0056, and *S*. Ealing CFS0080 were selected, as both were cultured from clinical sources. The whole genome sequences of *S*. Anatum and *S*. Ealing were obtained using the MiSeq platform. Using these data, comparison of the genomes could provide additional insights in to these historical bacterial isolates, the basic statistics of the genome assemblies from this study are shown in Table 1; *S*. Anatum CFS0056 and *S*. Ealing CFS0080 displayed the typical *Salmonella* DNA characteristics.

**Table 1:** **Comparitive features of the genomes of ***S***. Anatum and ***S***. Ealing with ***S***. Typhimurium ST4/74**.

**Features/*Salmonella* serovars**	**Typhimurium ST4/74**	**Anatum CFS0056**	**Ealing CFS0080**
Accession number	NC_016857	–	–
SRA number		SRP081283	SRP081283
Assembly contigs	n/a	71	51
Assembly N50	n/a	486,442	408,530
Genome size (Mbp)	4.87	4.87	4.82
%GC	52.18	52.42	52.17
Predicted coding sequences (CDS)	4782	4563	4471
No. of rRNA operons	22	9	10
No. of tRNA operons	85	78	83
No. of tmRNA operons	1	1	1

**Figure 2:**
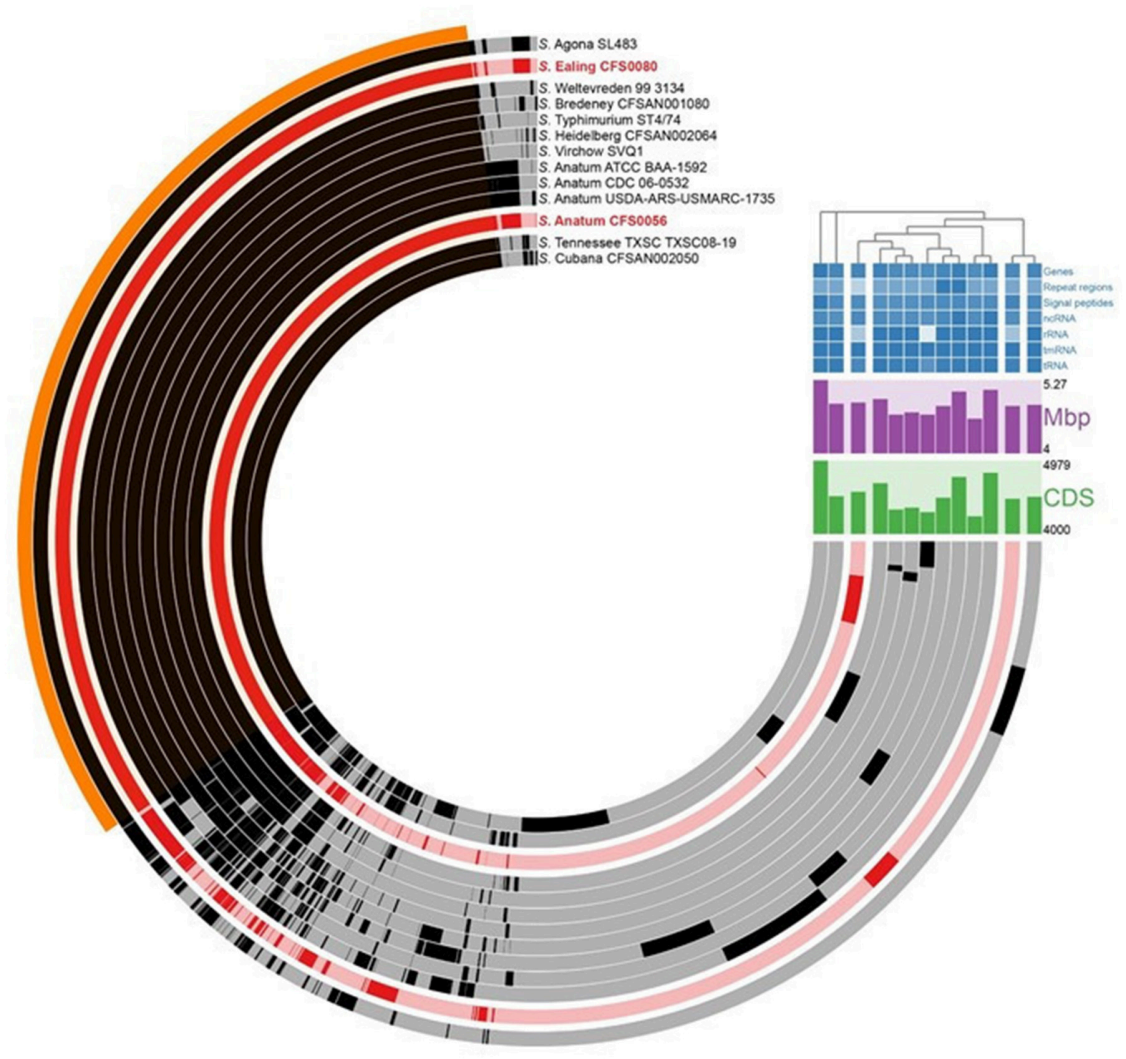
**Comparitive Anvi'o phylogenetic analysis of core genome from selected ***Salmonella*** serovars compared against ***S***. Anatum CFS0056 and ***S***. Ealing CFS0080**. Regions of the circular map shown in black denote similar content between isolates. Red colored regions shown in the circular map denote the genomes of the *S*. Anatum CFS0056 and *S*. Ealing CFS0080 study isolates. The region of the map marked in orange represents the core genome across all isolates. The coding sequence content (denoted as CDS; Green); the genome sizes (denoted as Mbp; Purple, see also Table 1) and a cluster diagram are also shown.

Availing of the Roary pipeline and Anvi'o visualization tool for broad genomic comparison, the core and accessory genomes for both serovars can be determined and then compared to a series of *Salmonella* isolates as shown in Figure 2. *Salmonella* Anatum CFS0056 and *S*. Ealing shared ~43% of all unique protein clusters identified (numbering 3544 of 8255) when compared against other selected isolates of this bacterial genus (Figure 2). A phylogenetic analysis using a core genome alignment of various *Salmonella* serovars showed that *S*. Ealing CFS0080 and *S*. Agona SL483 displayed a high degree of genetic similarity. *Salmonella* Agona SL483 was isolated in 2008 and epidemiologically linked to cases of food-borne human infections associated with the consumption of contaminated dried cereal foods (Fricke et al., 2011). In contrast *S*. Anatum CFS0056 from this study shared a high degree of similarity with other Anatum serovar isolates, although 260 protein clusters were unique to *S*. Anatum CFS0056 among the isolates included in this analysis. Overall, *S*. Ealing and *S*. Anatum shared seven unique proteins in comparison to the other isolates included in the analysis including TU1 elongation factor 1 along with hypothetical proteins.

### Comparative analysis of selected *Salmonella* pathogenicity islands (SPI) from the genomes of serovars anatum and ealing

**Figure 3:**
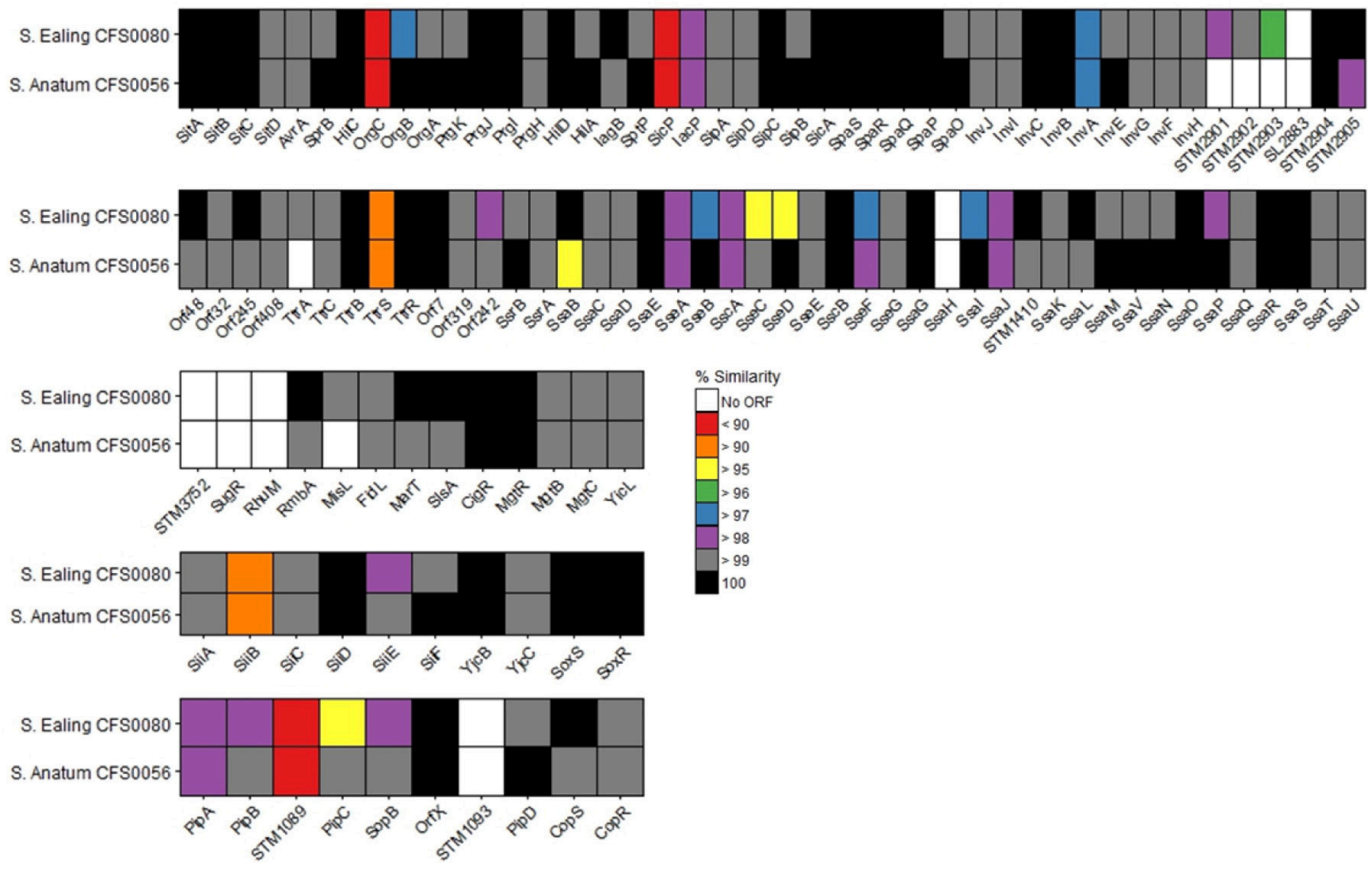
**Percentage similarity of SPI proteins 1–5 detected within ***S***. Anatum CFS0056 and ***S***. Ealing CFS0080 compared against reference strain ***S***. Typhimurium ST4/74**.

SPIs are horizontally acquired genetic cassettes that play a major role in *Salmonella* survival and virulence. The SPI-containing proteins from *S*. Anatum CFS0056 and *S*. Ealing CFS0080 appear to share high levels of similarity (ranging from < 95 to 100%) when compared against the reference *S*. Typhimurium ST4/74 (Figure 3). Proteins from SPI-1, -2, -4, and -5 display varying degrees of diversity, in comparison to SPI-3 which appears to be highly conserved. Upon closer inspection, *S*. Ealing CFS0080 demonstrated an overall lower sequence similarity in comparison to that observed for *S*. Anatum CFS0056 from this study. A total of 11 proteins were not detected from this analysis in one or both strains, a feature that should be interpreted with caution.

### Intracellular survival of *S. enterica* serovars in human macrophages

To study the ability of these two isolates to survive phagocytosis, *ex vivo* infections were performed using differentiated human THP-1 macrophages in a gentamicin protection assay. *Salmonella* Typhimurium ST4/74 was included as a reference strain. Infections were carried out at a Multiplicity Of Infection (MOI) of 10:1. Viable internalized bacteria were enumerated at 2, 4, 8, and 24 Hours Post Infection (HPI). Of the two serovars Anatum CFS0056 and Ealing CFS0080 tested in this study, all were found to persist within THP-1 macrophages for a period of up to 24 HPI. Although, the number of *S*. Ealing colonies recovered at 24 HPI was lower in comparison to *S*. Anatum and *S*. Typhimurium, pairwise comparison using ANOVA indicates this difference was statistically significant (*p* = 0.02).

## Discussion

PIF can become contaminated with bacteria of importance to human health, some of which may be pathogenic (including *Salmonella* species among others; Mullane et al., 2007). It is a constant challenge for the PIF industry generally, to be able to detect Class A pathogens such as *Salmonella* species in this food matrix prior to distribution due to the low numbers that are present on occasion. Failure to do so, can lead to salmonellosis infection among infants, some of which may be life-threatening (Cahill et al., 2008). Similarly, failures in the food safety management of these associated food production facilities can result in the environment and final product becoming contaminated. This retrospective study investigated *Salmonella* serovars Anatum and Ealing cultured from two historical outbreaks and compared their molecular characteristics.

Molecular fingerprinting identified two clonal clusters consistent with the two serovars, Anatum and Ealing. Overall both serovars displayed a high degree of genetic diversity with profiles sharing similarities ranging from 85 to 100%. The majority of *S*. Ealing produced pulsotypes that were indistinguishable using *Xba*1 or *Spe*1 alone (Figure 1), with the exception of isolate CFS0091, which was determined to be un-related to the outbreak. Comparative analysis of the pulsotypes obtained for *S*. Anatum isolates CFS0048-0050 and CFS0052-0056 (Figure 1), suggested that these isolates of clinical origin and from the PIF production environment were linked during the 1996/1997 outbreak. Whole genome sequencing was performed on *S*. Anatum CFS0056 and *S*. Ealing CFS0080. These isolates were selected as representatives due to their apparent clonal nature and clinical isolation.

Analysis of the core genome revealed *S*. Anatum CFS0056 and *S*. Ealing CFS0080 share ~43% of the unique protein clusters identified by Prokka annotation. In this study *S*. Anatum was found to be more homologous when compared with other representatives of the same serovar, though with some unique regions being identified (Figure 2). These unique genomic loci could be related to the survival phenotype of this isolate in the PIF production environment and therefore require further exploration. Moreover, *S*. Anatum is not typically associated with milk-related outbreaks but is more commonly encountered in meat-processing settings (Ebuchi et al., 2006; Marasini et al., 2016). In the analysis displayed here *S*. Anatum also demonstrated a distant relationship to *S*. Typhimurium together with the *Salmonella* serovars Virchow and Heidelberg. Both of these latter isolates were previously cultured from outbreaks involving vegetable and meat related products. When analyzed, *Salmonella* Ealing was found to cluster only with *S*. Agona SL483 a pathogen also implicated in a food-related outbreak linked dried cereal food product, suggesting the two isolates have similar genetic elements which could contribute to survival in low moisture environments.

Comparative analysis at the level of the amino acid sequence of SPI-1 through to SPI-5 was performed as these are the most common SPIs in all *Salmonella enterica* serovars sequenced to date. These SPIs code for virulence factors which facilitate internalization of the pathogen and have an impact on the survival of the bacterium in the host intercellular environment. Of specific interest were SPI-1 and -2 that encode genes that function in bacterial virulence including adhesion, and vacuole stabilization. Figure 3 presents the comparison in the form of a heat map that shows a near complete complement of genes was detected across all isolates for SPI-1–SPI-5 together with a high level of amino acid similarity being observed between the outbreak and reference strains. This comparative approach also identified proteins that are more diverse when compared to the reference, including those with key roles in intercellular survival and virulence (reviewed in McGhie et al., 2009; Fricke et al., 2011; Ruby et al., 2012; Lathrop et al., 2015). Proteins located within SPI-1, -2, -4, and -5 showed the greatest diversity and these in particular included proteins critical in infection and persistence such as SiiEFG that functions in bacterial adhesion and invasion, SseFG, SopB, which are essential in *Salmonella* containing vacuole stabilization and replication and PipABC which are involved in entropathogenicity (Wood et al., 1998; Morgan et al., 2007; Ibarra and Steele-Mortimer, 2009). SPI-3 showed a high degree of similarity with the reference strain. Of note, three proteins (RhuM, SugR, STM3752), were not detected in this analysis, an observation that does not necessarily mean these genes were absent.

Overall *S*. Ealing CFS0080 displayed a greater degree of sequence diversity across the SPI island proteins in comparison to *S*. Anatum CFS0056, when compared to *S*. Typhimirium ST4/74. The consequences of sequence divergence within the SPI genes is not clear at present but targeted knockout studies of selected genes across SPIs 1–5 suggest that if pseudogenes accumulate in the bacterial genome, an impaired virulence phenotype could result (Rychlik et al., 2009). This particularly relates to SPI-1 and SPI-2 which are essential for host adaption and systemic infection. If these two SPIs become non-functional the virulence and survival of the strains, in the human host could be impaired at the level of the phenotype (Ibarra and Steele-Mortimer, 2009).

**Figure 4:**
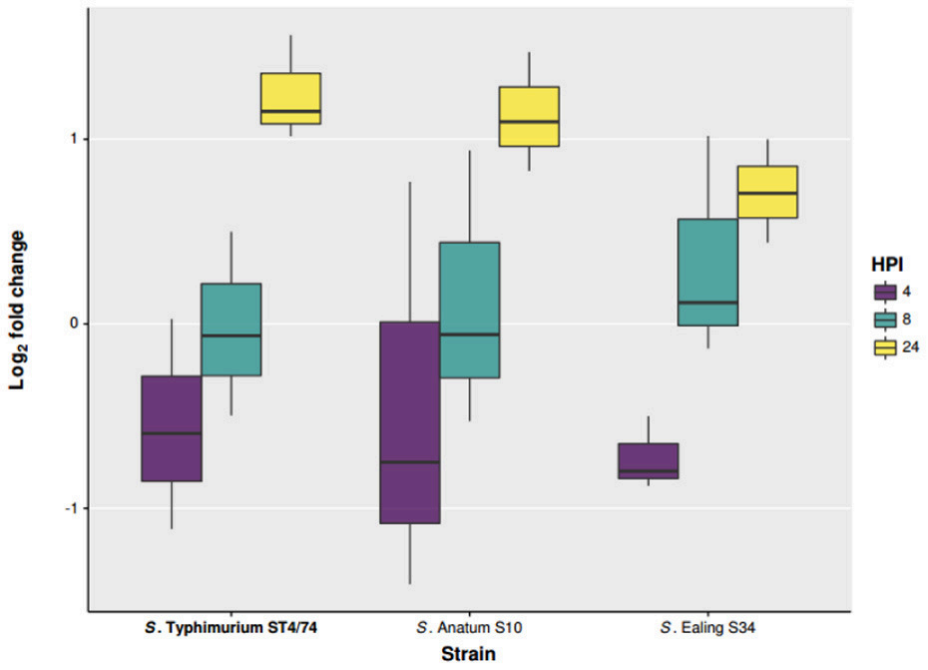
**Intercellular survival and proliferation of ***S***. Anatum CFS0056, ***S***. Ealing CFS0080, and ***S***. Typhimurium ST4/74 in THP-1 human macrophage cell lines**.

Both *S*. Anatum CFS0056 and *S*. Ealing CFS0080 survived for 24 HPI in human THP-1 macrophages (Figure 4). The numbers of bacteria recovered after 4 and 8 HPI were comparable to those of *S*. Typhimurium ST4/74. However, after 24 HPI, the cell count for *S*. Ealing CFS0080 was found to be less than that of *S*. Anatum CFS0056 and *S*. Typhimurium ST4/74. The adaption of *Salmonella* to the stresses experienced in the host-macrophage environment has been associated with the development of viable non-replicating cells (Helaine et al., 2010, 2014). Further, another report has also observed the potential link between reduced virulence and increased SPI variability using cell infection models (McWhorter and Chousalkar, 2015). The variation in sequence conservation across SPI proteins could be associated with non-functioning proteins leading to attenuated *in-vivo* survival.

## Conclusion

In this study, the molecular characteristics of *S*. Anatum and *S*. Ealing cultured from powdered infant formula related outbreaks were studied. Pulsotyping with PFGE demonstrated the clonal nature of the outbreak strains and this feature facilitated their distinction from unrelated isolates of the same serovar. WGS and subsequent analysis of selected representatives of these two serovars identified genomic regions that were common, but also showed that a large accessory genome could be identified. Macrophage infection studies confirmed the ability of both serovars to persist in the host cell for similar periods but at different bacterial cell numbers, reflecting differences in sequence conservation among the SPI islands studied.

Overall the results from this study provide a useful foundation to begin to extend the investigation of how these unique bacterial isolates adapted to the PIF-processing environment, a necessary prelude to the outbreaks reported in each case.

## Author contributions

LG—drafted the manuscript (MS), collated, and reviewed all results and contributed to the WGS and subsequent bioinformatics analysis. SFi—assembled the collection of isolates and performed the initial geno- and phenotypic-studies whilst contributing to the drafting of the MS. DH—carried out cell culture work; evaluated all of the WGS data and carried to the bioinformatics analysis. LB—performed cell culture work. EW—performed phenotype characterization of the isolates. CI—assembled the collection of isolates and performed the initial geno- and phenotypic-studies whilst contributing to the drafting of the MS. JT—provided bacteria and background information. SFa—co-ordinated the study.

### Conflict of interest statement

The authors declare that the research was conducted in the absence of any commercial or financial relationships that could be construed as a potential conflict of interest.

## Footnotes

^1^danieljhurley/atypical-Salmonella. GitHub Available online at: https://github.com/danieljhurley/atypical-Salmonella (Accessed August 10, 2016).

## References

[B1] Bankevich A. Nurk S. Antipov D. Gurevich A. A. Dvorkin M. Kulikov A. S.. (2012). SPAdes: a new genome assembly algorithm and its applications to single-cell sequencing. J. Comput. Biol. 19, 455–477. 10.1089/cmb.2012.0021 22506599 PMC3342519

[B2] Bolger A. M. Lohse M. Usadel B. (2014). Trimmomatic: a flexible trimmer for Illumina sequence data. Bioinformatics 30, 2114–2120. 10.1093/bioinformatics/btu170 24695404 PMC4103590

[B3] Brouard C. Espié E. Weill F. X. Kérouanton A. Brisabois A. Forgue A. M.. (2007). Two consecutive large outbreaks of *Salmonella enterica* serotype Agona infections in infants linked to the consumption of powdered infant formula. Pediatr. Infect. J. 26, 148–152. 10.1097/01.inf.0000253219.06258.23 17259878

[B4] Cahill S. M. Wachsmuth I. K. Costarrica M. de L. Embarek P. K. B. (2008). Powdered infant formula as a source of *Salmonella* infection in infants. Clin. Infect. Dis. 46, 268–273. 10.1086/524737 18171262

[B5] Ebuchi S. Baba A. Uryu K. Hiwaki H. (2006). Two outbreaks caused by *Salmonella* Derby and *S*. Anatum at grilled-meat restaurants in Fukuoka city. Jpn. J. Infect. Dis. 59, 405–406. 17186965

[B6] Eren A. M. Esen Ö. C. Quince C. Vineis J. H. Morrison H. G. Sogin M. L.. (2015). Anvi'o: an advanced analysis and visualization platform for ‘omics data. PeerJ 3:e1319. 10.7717/peerj.1319 26500826 PMC4614810

[B7] Fricke W. F. Mammel M. K. McDermott P. F. Tartera C. White D. G. Leclerc J. E.. (2011). Comparative genomics of 28 *Salmonella enterica* isolates: evidence for CRISPR-mediated adaptive sublineage evolution. J. Bacteriol. 193, 3556–3568. 10.1128/JB.00297-11 21602358 PMC3133335

[B8] Helaine S. Cheverton A. M. Watson K. G. Faure L. M. Matthews S. A. Holden D. W. (2014). Internalization of *Salmonella* by macrophages induces formation of nonreplicating persisters. Science 343, 204–208. 10.1126/science.1244705 24408438 PMC6485627

[B9] Helaine S. Thompson J. A. Watson K. G. Liu M. Boyle C. Holden D. W. (2010). Dynamics of intracellular bacterial replication at the single cell level. Proc. Natl. Acad. Sci. U.S.A. 107, 3746–3751. 10.1073/pnas.1000041107 20133586 PMC2840444

[B10] Ibarra J. A. Steele-Mortimer O. (2009). *Salmonella* – the ultimate insider. *Salmonella* virulence factors that modulate intracellular survival. Cell. Microbiol. 11, 1579–1586. 10.1111/j.1462-5822.2009.01368.x 19775254 PMC2774479

[B11] Lathrop S. K. Binder K. A. Starr T. Cooper K. G. Chong A. Carmody A. B.. (2015). Replication of *Salmonella enterica* serovar typhimurium in human monocyte-derived macrophages. Infect. Immun. 83, 2661–2671. 10.1128/IAI.00033-15 25895967 PMC4468555

[B12] Li H. (2015). BFC: correcting Illumina sequencing errors. Bioinformatics 31, 2885–2887. 10.1093/bioinformatics/btv290 25953801 PMC4635656

[B13] Marasini D. Abo-Shama U. H. Fakhr M. K. (2016). Complete genome sequences of *Salmonella enterica* serovars anatum and anatum var. 15+, isolated from retail ground turkey. Genome Announc. 4:e01619–15. 10.1128/genomeA.01619-15 26798111 PMC4722278

[B14] McGhie E. J. Brawn L. C. Hume P. J. Humphreys D. Koronakis V. (2009). *Salmonella* takes control: effector-driven manipulation of the host. Curr. Opin. Microbiol. 12, 117–124. 10.1016/j.mib.2008.12.001 19157959 PMC2647982

[B15] McWhorter A. R. Chousalkar K. K. (2015). Comparative phenotypic and genotypic virulence of *Salmonella* strains isolated from Australian layer farms. Food Microbiol. 6:12. 10.3389/fmicb.2015.00012 25667583 PMC4304256

[B16] Morgan E. Bowen A. J. Carnell S. C. Wallis T. S. Stevens M. P. (2007). SiiE is secreted by the *Salmonella enterica* Serovar typhimurium pathogenicity island 4-encoded secretion system and contributes to intestinal colonization in cattle. Infect. Immun. 75, 1524–1533. 10.1128/IAI.01438-06 17220313 PMC1828598

[B17] Mullane N. R. Iversen C. Healy B. Walsh C. Whyte P. Wall P. G.. (2007). Enterobacter sakazakii an emerging bacterial pathogen with implications for infant health. Minerva Pediatr. 59, 137–148. 17404564

[B18] Needleman S. B. Wunsch C. D. (1970). A general method applicable to the search for similarities in the amino acid sequence of two proteins. J. Mol. Biol. 48, 443–453. 10.1016/0022-2836(70)90057-4 5420325

[B19] Page A. J. Cummins C. A. Hunt M. Wong V. K. Reuter S. Holden M. T. G.. (2015). Roary: rapid large-scale prokaryote pan genome analysis. Bioinformatics 31, 3691–3693. 10.1093/bioinformatics/btv421 26198102 PMC4817141

[B20] Ribot E. M. Fair M. A. Gautom R. Cameron D. N. Hunter S. B. Swaminathan B.. (2006). Standardization of pulsed-field gel electrophoresis protocols for the subtyping of *Escherichia coli* O157: H7, *Salmonella*, and Shigella for PulseNet. Foodbourne Pathog. Dis. 3, 59–67. 10.1089/fpd.2006.3.59 16602980

[B21] Rowe B. Begg N. T. Hutchinson D. N. Dawkins H. C. Gilbert R. J. Jacob M.. (1987). *Salmonella* ealing infections associated with consumption of infant dried milk. Lancet 2, 900–903. 10.1016/S0140-6736(87)91384-5 2889093

[B22] Ruby T. McLaughlin L. Gopinath S. Monack D. (2012). *Salmonella*'s long-term relationship with its host. FEMS Microbiol. Rev. 36, 600–615. 10.1111/j.1574-6976.2012.00332.x 22335190

[B23] Rychlik I. Karasova D. Sebkova A. Volf J. Sisak F. Havlickova H.. (2009). Virulence potential of five major pathogenicity islands (SPI-1 to SPI-5) of *Salmonella enterica* serovar Enteritidis for chickens. BMC Microbiol. 9:268. 10.1186/1471-2180-9-268 20021686 PMC2803193

[B24] Seemann T. (2014). Prokka: rapid prokaryotic genome annotation. Bioinformatics 30, 2068–2069. 10.1093/bioinformatics/btu153 24642063

[B25] Threlfall E. J. Ward L. R. Hampton M. D. Ridley A. M. Rowe B. Roberts D.. (1998). Molecular fingerprinting defines a strain of *Salmonella enterica* serotype Anatum responsible for an international outbreak associated with formula-dried milk. Epidemiol. Infect. 121, 289–293. 10.1017/S0950268898001149 9825779 PMC2809525

[B26] Wood M. W. Jones M. A. Watson P. R. Hedges S. Wallis T. S. Galyov E. E. (1998). Identification of a pathogenicity island required for *Salmonella* enteropathogenicity. Mol. Microbiol. 29, 883–891. 10.1046/j.1365-2958.1998.00984.x 9723926

[B27] Zheng J. Keys C. E. Zhao S. Meng J. Brown E. W. (2007). Enhanced subtyping scheme for *Salmonella* enteritidis. Emer. Infect. Dis. 13:1932. 10.3201/eid1312.070185 18258051 PMC2876743

