# Corrigendum: Molecular Characterization of *Salmonella* Serovars Anatum and Ealing Associated with Two Historical Outbreaks, Linked to Contaminated Powdered Infant Formula

**DOI:** 10.3389/fmicb.2017.00015

**Published:** 2017-01-20

**Authors:** Lynda Gunn, Sarah Finn, Daniel Hurley, Li Bai, Ellen Wall, Carol Iversen, John E. Threlfall, Séamus Fanning

**Affiliations:** ^1^UCD-Centre for Food Safety, School of Public Health, Physiotherapy and Sports Science, University College DublinDublin, Ireland; ^2^Key Laboratory of Food Safety Risk Assessment, Ministry of Health, China National Center for Food Safety Risk AssessmentBeijing, China; ^3^Health Protection AgencyLondon, UK; ^4^Institute for Global Food Security, Queen's University BelfastBelfast, UK

**Keywords:** PFGE, whole genome sequencing, *Salmonella* pathogenicity islands, core genome, infection model

There was a mistake in the strain names of Figure 4 as published. The correct version of Figure 4 appears below. The authors apologize for the mistake. This error does not change the scientific conclusions of the article in any way.

**Figure 4:**
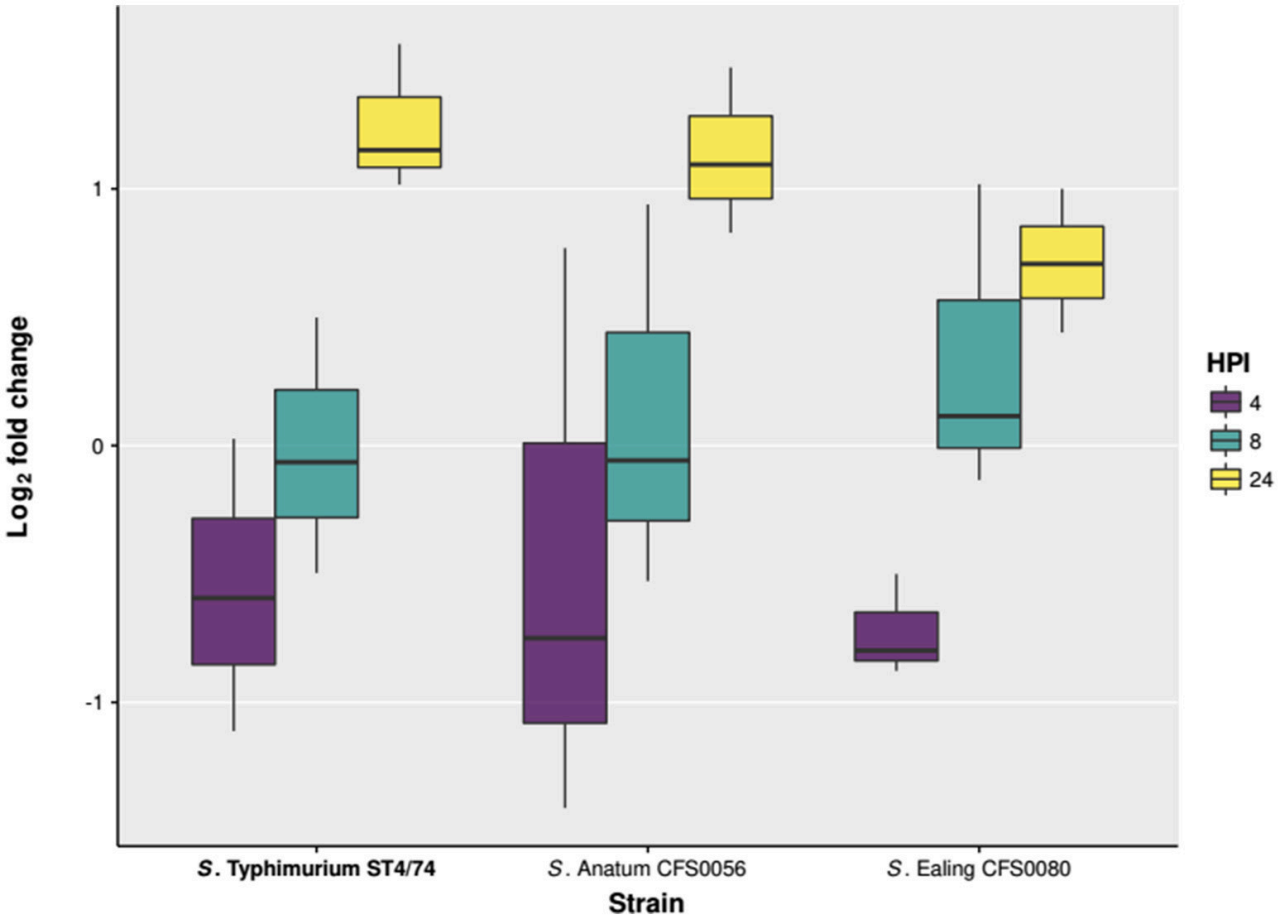
**Intercellular survival and proliferation of ***S***. Anatum CFS0056, ***S***. Ealing CFS0080, and ***S***. Typhimurium ST4/74 in THP-1 human macrophage cell lines**.

## Conflict of interest statement

The authors declare that the research was conducted in the absence of any commercial or financial relationships that could be construed as a potential conflict of interest.

